# Examining the influence of shyness on children’s helping and comforting behaviour

**DOI:** 10.3389/fpsyg.2023.1128588

**Published:** 2023-02-27

**Authors:** Tara A. Karasewich, Cameron Hines, Sylvia G. V. Pinheiro, Nina Buchenrieder, Kristen A. Dunfield, Valerie A. Kuhlmeier

**Affiliations:** ^1^Department of Psychology, Queen’s University, Kingston, ON, Canada; ^2^Department of Psychology, Concordia University, Montreal, QC, Canada

**Keywords:** shyness, helping, comforting, social cognition, moral development, prosocial behaviour, methodology, individual differences

## Abstract

**Introduction:**

Shy children, who tend to feel anxious around others and withdraw from social interactions, are found to be less prosocial than their not-shy peers in some studies, though not in others. To examine the contexts in which shy children may be more or less likely to engage in prosocial behaviour, we compared children’s willingness and ability to intervene during in-person tasks that differed in *socialengagement demands* and *complexity*, factors that have been conflated in past research.

**Methods:**

We presented 42, 3.5- to 4.5-year-old children with prosocial problems that varied, in a 2 x 2 within-subjects design, by the type of intervention required (i.e., simple helping or complex comforting) and the source of the problem (i.e., social: within the experimenter’s personal space; or object: a target object distanced from her).

**Results:**

Most of the children acted prosocially, with little prompting, in the two helping tasks and in the object-centered comforting task. In contrast, fewer than half of the children acted prosocially in the social-centered comforting task. Shyer children were not less likely to intervene in any of the four tasks, but they were slower to intervene in the object-centred comforting task, in which the experimenter was upset about a broken toy.

**Discussion:**

Thus, providing social-centered comfort to a recently-introduced adult is challenging for young children, regardless of shyness, though shy children do show hesitancy with object-centered comforting. Further, these findings provide insights into the methodological challenges of disentangling children’s prosocial motivation and understanding, and we propose solutions to these challenges for future research.

## Introduction

The early emergence of *prosocial behaviour* (i.e., acting with the intent to benefit someone in need) has been well-established by developmental research (e.g., Radke-Yarrow et al., 1976; Eisenberg and Mussen, 1989; Warneken and Tomasello, 2006; Dunfield et al., 2011; Geraci and Franchin, 2021; Tavassoli et al., 2022). Children as young as 12 months have been observed to *help* others meet instrumental goals, and older preschoolers have been found to *share* material resources, *comfort* those in emotional distress, and *cooperate* with others to meet joint goals (Liszkowski, 2005; Warneken and Tomasello, 2007; Svetlova et al., 2010; Dunfield and Kuhlmeier, 2013). Yet, less is known about possible individual differences in children’s prosocial interventions (e.g., Pettygrove et al., 2013; Imuta et al., 2016; Chernyak et al., 2018). For example, *shy* children, who tend to feel anxious around others and withdraw from social interactions (Coplan and Armer, 2007), may be less prosocial than their not-shy peers, under certain conditions. As examples, shyer children have been found to intervene less often on behalf of an experimenter than their own mothers, to intervene less in socially engaged ways, and to require more prompting in order to intervene (e.g., Young et al., 1999; Beier et al., 2017; Karasewich et al., 2019; MacGowan and Schmidt, 2021). It is important to note, however, that shyness effects on young children’s prosocial behaviour have not been found in all studies (e.g., Schuhmacher et al., 2017; Grossmann et al., 2020).

In the current study, we examine the prosocial behaviour of shy children from two angles: the *motivation* to intervene on behalf of a person in need and the *understanding* of how to do so (Dunfield, 2014; Paulus, 2014; Martin and Olson, 2015; Eisenberg et al., 2016). Many related definitions of shyness have been used in the research literature (e.g., Buss, 1986; Henderson and Zimbardo, 2010; Hassan et al., 2021); in this study we consider shyness to be a tendency to feel anxious around others and withdraw from social interactions (Coplan and Armer, 2007). It is likely that shy children will be less willing to intervene whenever they are feeling anxious, and they may also struggle to process prosocial situations in order to effectively intervene (e.g., Young et al., 1999; MacGowan and Schmidt, 2021). Thus, we tested how preschool children ranging in shyness would respond to four prosocial tasks that varied in *social engagement demands* and *complexity*, which are two factors that have been conflated in past research (e.g., Beier et al., 2017; Karasewich et al., 2019). To foreshadow, the results of the present study subverted our expectations in interesting ways, which in turn allowed for a consideration of the methods currently used to study shyness and the early development of prosociality.

### Social engagement demands

Previous research suggests that positive emotions such as interest in others can support the production and development of prosocial behaviour (Hammond and Drummond, 2019). Yet, prosocial situations can vary greatly in the amount of social engagement they encourage from, or even require of, children who want to intervene. Many of the prosocial problems featured in laboratory studies could be considered *low* in social engagement demands because they allow children to intervene without drawing a lot of attention to themselves. For example, a child could help a person struggling to get an object that is out of their reach by just handing the object to them, without speaking to or coming into contact with anyone (e.g., Warneken and Tomasello, 2006; Dunfield and Kuhlmeier, 2013; Pettygrove et al., 2013). We would expect *shy* children to feel more motivated to act prosocially whenever social engagement demands are low as compared to when these demands are high (but see: MacGowan and Schmidt, 2021). This prediction is supported by Beier et al. (2017), in which shyer children readily helped an experimenter in a typical out-of-reach object task (i.e., picking up a pen that had fallen from her desk), but needed more prompting to help her in a highly social one (i.e., getting someone else’s attention). Similarly, Karasewich et al. (2019) found shy children to be less likely to help in an out-of-reach object task that was modified to be very socially demanding (i.e., asking an unfamiliar adult to get the experimenter’s toy from a high shelf).

In the present study, we categorized the social engagement demands of our tasks by whether or not they encouraged children to *approach* the experimenter while intervening. Thus, our highly demanding “social-centred” tasks consisted of problems within the experimenter’s personal space, while our less demanding “object-centred” tasks involved at least one object that was more distant from her. We also examined *how* children intervened, when they chose to do so. In particular, we expected shyer children to focus on objects while intervening (i.e., to be “object-oriented”) instead of focusing on the experimenter herself (i.e., “social-oriented”).

### Complexity of prosocial problems

Prosocial tasks can also vary in their complexity. For a child to intervene effectively, they must first understand what problem the person in need is having and then come up with a solution that they could, realistically, enact (Dunfield, 2014). *Helping* others to meet their instrumental goals tends to be a simple task for young children. Most helping problems have one clear solution, with any variability in response constrained by the goal needing to be met. Take, for example, a teacher who needs a book from the far side of their desk: a child could help by *either* handing it over or pushing it closer, but both actions enact the same “retrieve book” solution. In contrast, there are many potential ways that a child could *comfort* someone experiencing emotional distress (e.g., reassuring them it will be okay, giving them a hug, fixing something broken, etc.), so there is no uniquely “correct” or “obvious” response to that type of problem. Children must also be able to recognize when another person is upset before they can provide comfort, and this ability emerges later in development than the goal-understanding required for helping (Hoffman, 1982; Sommerville and Woodward, 2005; Rosenqvist et al., 2014; see also Paulus et al., 2013). It is unsurprising, then, that preschool children have been found to more readily help others than comfort them in lab settings (e.g., Radke-Yarrow et al., 1976; Svetlova et al., 2010; Dunfield and Kuhlmeier, 2013). Complex prosocial problems may be especially difficult for shy children, who have been found to differ in how they process social situations, more broadly, and in what they understand of others’ mental states (e.g., LoBue and Pérez-Edgar, 2014; Gal-Szabo et al., 2017). We explored this possibility in our study by comparing children’s responses in helping and comforting tasks.

Yet, the distinction we have just made between helping and comforting is actually oversimplified. Not all helping problems have a solution that would be obvious to young children. In Karasewich et al.’ (2019) study, for example, there was no way that a child could help the experimenter on their own. Because the experimenter’s toy was on a high shelf, children could only help her *indirectly*, by first determining who could reach the shelf and then asking that person to intervene. Recognizing *why* someone needs help can also be fairly complex. To understand why the experimenter in Beier et al.’ (2017) social helping task was failing to get someone’s attention, children had to recognize that she could not raise her voice (she was speaking in a raspy whisper) and that the other person was not looking at her. Both of these helping tasks could be considered more complex than the ones that have been directly compared to comforting tasks in past research (e.g., Dunfield et al., 2011; Chernyak et al., 2018). They are also, as highlighted above, both higher in social engagement demands, which makes interpreting their findings more difficult. We cannot know whether the shyness effects found in these studies were the result of shy children being reluctant to intervene in highly social ways, having trouble figuring out what to do, or some combination of both. Here, we designed our prosocial tasks to vary *systematically* in social engagement demands and complexity. Thus, our social-centred helping problem was made to be just as simple as our object-centred one, while our comforting problems were meant to be more obscure.

### The present study

To summarize, in this study we examined 3.5- to 4.5-year-old children’s willingness and ability to respond prosocially in four within-subjects tasks that varied in social engagement demands (i.e., “object-centred” vs. “social-centred” problems) and complexity (i.e., simple helping vs. more complex comforting problems). We predicted that shyer children would intervene more frequently and spontaneously in the object-centred tasks than the social-centred tasks. Further, we predicted that all children would find it easier to help the experimenter than to comfort her, but that this would be especially true of shyer children. Finally, we expected shyer children who did intervene to use fewer “social-oriented” strategies and more “object-oriented” ones. Thus, our systematic approach to varying the social demands and complexity of our prosocial tasks allowed us to examine individual differences in children’s motivation to intervene and understanding of how to do so. As noted above, this approach yielded unexpected results that provided the opportunity for nuanced consideration of the methods by which developmental researchers study the relationship between shyness and prosocial behaviour.

## Methods

### Participants

Testing occurred in the Social Cognition laboratory at Queen’s University. Participants were 42 preschool children (19 male; 23 female), with an average age of 47.7 months (range: 42–54 months). This age range was chosen in order to examine children during a point in development in which they typically begin to provide direct comfort more regularly (e.g., Svetlova et al., 2010; Dunfield et al., 2011). Nine additional children were tested but excluded from the final analysis due to experimenter error (4), equipment failure (1), and participant factors (4); for further detail, see Supplementary Table S1 on our Open Science Framework page. Sample size was assessed through a post-hoc power analysis, using G*Power 3.1.9.2 (Faul et al., 2007). With an *alpha* of 0.05 and an effect size of 0.36, which was found in a prior study assessing the relation between shyness and comforting in preschool children (Young et al., 1999), power was calculated to be 0.79 for our sample size. We found similar results by plotting a sensitivity power curve (see Supplementary Figure S1). Families were recruited from Kingston, a small city in Canada that has a predominantly White and middle-class population, and participants were representative of the region. Data was collected prior to the COVID-19 pandemic. All children were given a small gift at the end of the study to thank them for participating. This study was conducted with approval from Queen’s General Research Ethics Board.

### Procedure

#### Attachment Q-Sort and the shyness subscale

While children participated in the experimental paradigm, their mothers completed the Attachment Q-Sort in a separate room (computerized version 2.1.2: Waters, 1995; Soria, 2015), which we then used to create a shyness subscale. A research assistant explained the Q-Sort instructions and provided help when requested, but the task was otherwise completed independently. On a computer, mothers were shown 90 cards with statements describing a young child’s behaviour (e.g., “Child is lighthearted and playful most of the time”; “Child easily becomes angry with toys”) and were asked to create nine piles of 10 cards each, from *most uncharacteristic* of their child (pile 1) to *most characteristic* (pile 9; see Supplementary Figure S2). At the end of the sort, mothers were asked to review and confirm their choices.

Although the Q-Sort is a measure of attachment security (which we do calculate in the online supplement), we primarily used it to create an *ad-hoc* “shyness” subscale. Following the procedure described by Waters (n.d.), three of the study authors first identified and reached consensus on 14 items from the original 90-item scale that describe the child behaving withdrawn around other people (e.g., “Child runs to mother with a shy smile when new people visit the home”) or the opposite (e.g., “Child laughs and smiles easily with a lot of different people”), the latter of which were reverse-coded. We then examined the relation between each pair of items within our sample and removed 5 items that had a low number of significant inter-item correlations. The internal consistency of the subscale was improved by removing each of these items, until we were left with a 9-item subscale that had high internal consistency (Cronbach’s *alpha* = 0.810). A shyness score for each child was calculated by summing the mother’s sort-value (i.e., 1–9) for each subscale item, with higher scores indicating that the child displays shy behaviours more often than not. Supplementary Table S2 lists the 9 items that were used in the final subscale and the 5 items that were removed. Although we did not look at Waters’ (1987) original rationale for each Q-sort item when creating the shyness subscale, it is interesting to note that he considered 11 of the 14 items we tested (6 of which were included in the final subscale) to be items that either mask the purpose of the Attachment Q-Sort from raters or discriminate between attachment security and temperament.

#### Prosocial tasks

In the testing room, children completed four prosocial tasks that varied in a 2 (type: help, comfort) x 2 (source of the problem: object, social) design. Helping tasks were characterized by instrumental need: the experimenter had a goal that she could not complete on her own. Comforting tasks were characterized by emotional distress: she was upset after an unfortunate event. In social-centred tasks, the source of the experimenter’s problem was within her personal space, so the child would have to focus on (and potentially come in contact with) her to respond effectively. In object-centred tasks, her problem involved a target object (s) that was outside of her personal space, allowing the child to respond while focusing solely on the object (s). The order of the prosocial tasks (see Supplementary Tables S3, S4) was counterbalanced, with approximately half of the sample receiving a helping task first (*n* = 22) and half receiving a comforting task first (*n* = 20). Two individuals acted as the experimenter for approximately half of the sample each (i.e., 23 vs. 19 participants).

**Figure 1:**
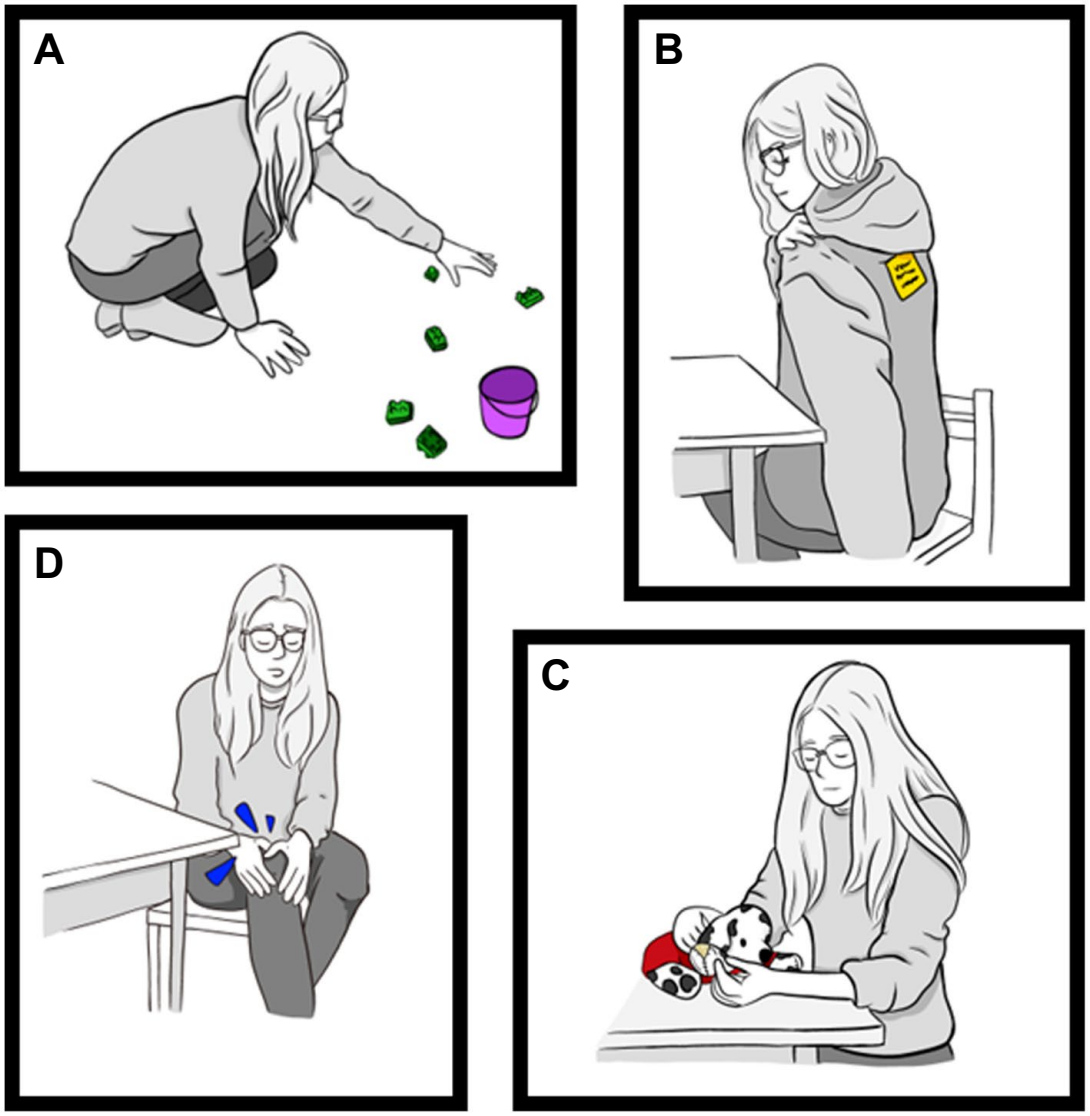
The four prosocial problems. In the object-centred helping task **(A)**, the experimenter spilled a bucket of Lego bricks. In the social-centred helping task **(B)**, she struggled to reach a sticker on the back of her jacket. In the object-centred comforting task **(C)**, she showed distress after discovering a rip in the leg of her favourite toy. In the social-centred comforting task **(D)**, she hit her knee on a doorframe and sat down in pain. Illustrations by Sylvia Pinheiro.

Figure 1 depicts the experimenter’s problem in each of the four prosocial tasks. In the object-centred helping task, she “accidentally” spilled a bucket of Lego bricks onto the floor. In the social-centred helping task, she “realized” there was a sticker on the back of her jacket that she could not reach. In the object-centred comforting task, she became upset after she “noticed” a rip in the leg of her favourite toy dog. Finally, in the social-centred comforting task, she “hurt” herself by banging her knee on the doorframe of an adjoining room. Within each task, the experimenter gave up to three cues to prompt the child to respond, spaced approximately 5 s apart for a total of 15 s. She always began by stating her problem (cue 1), then provided detail to clarify her problem (cue 2), and finally asked the child directly if they could intervene (cue 3). Each task ended if the child performed a prosocial act at the highest level of engagement (i.e., picking up the target objects in the helping tasks and approaching the experimenter to provide physical comfort in the comforting tasks). In all four tasks, the experimenter thanked the child after they intervened. When a child started to help in the object-centred helping task, she moved away to let them pick up the rest of the bricks alone, under the pretense that she had to put away another toy. We describe the full task procedures in Supplementary Table S5.

#### Delay games

The experimenter and child played four “delay” games in between the prosocial tasks, to give them a more naturalistic appearance. The first game, which acted as a warm-up period for the child, involved completing a puzzle. On average, children spent 6.15 min completing the puzzle with the experimenter (range: 3.08–10.90 min). In the second game they built a tower together out of the same Lego bricks that would then be used in the object-centred helping task. The third game was “Memory,” in which the child and experimenter took turns picking animal cards laid upside-down on the table to find matching pairs. The final game was two or three rounds of “tic-tac-toe,” in which they took turns putting their symbol on a 3×3 grid to make a line of 3 in a row.

#### Coding and interrater analyses

Coding for this study was completed at two separate time-points: before and after we pre-registered the analysis plan on OSF. Video recordings of the test sessions were transcribed and all behaviours were coded by two independent raters who overlapped on over 25% of the sample, which was used to calculate interrater reliability/agreement. Disagreements in ratings were resolved by the principal investigator. We describe additional coding used for exploratory analyses in the online supplement.

Responses to the prosocial tasks were categorized on ordinal scales of increasing engagement with the experimenter. The levels of each scale are summarized in Table 1, below. There were three categories of non-prosocial responses in all four tasks. First, at the lowest level of engagement, a child could not respond at all (e.g., watching the experimenter without doing or saying anything). The next level of engagement, an “empty response,” indicated that the child spoke during the task but did not address the experimenter’s problem (e.g., “I see puppets!”; “What is this [marking] on the table?”). Finally, a “concerned response” indicated that the child spoke to the experimenter about her problem but did not offer a solution (e.g., “I know those kinds of things, they hurt really bad”; “Are you upset?”). A concerned response (a form of *hypothesis testing*: Zahn-Waxler et al., 1992) can be considered one step away from prosocial behaviour – the child recognizes that something has happened to the person in need, but may not understand *what* their problem is or how to intervene (e.g., Knafo et al., 2008; Liew et al., 2011; MacGowan and Schmidt, 2021).

**Table 1:** Coding level of engagement in the prosocial tasks.

	Response type	Lego	Sticker	Broken toy	Hurt knee
Non-prosocial responses	No response	0	0	0	0
	Empty response	1	1	1	1
	Concerned response	2	2	2	2
Prosocial responses	Verbal help/comfort	3	3	3	3
	Physical help: Pick up bricks	3	--	--	--
	Physical comfort: Fix toy	--	--	3	--
	Physical help/comfort: Approach experimenter	--	4	4	4

“Prosocial responses” varied by task but always required that the child try to solve the experimenter’s problem. In all four tasks, the lowest level of prosocial response was to provide verbal help or comfort (e.g., “It will feel better in a few days”; “You can tape it”). The highest level of prosocial response for three of the tasks was to approach the experimenter to physically solve her problem: in the social-centred helping task, children could take the sticker off of the experimenter’s jacket, in the object-and social-centred comforting tasks they could give her a hug or other form of physical affection, and in the social-centred comforting task they could also kiss or rub her knee to make it feel better. The object-centred helping task did not have an equivalent level of engagement; physically helping the experimenter by picking up (at least some) of the spilled Lego bricks did not involve approaching her, so it was categorized at the same level of engagement as verbal helping. Similarly, trying to fix the broken toy in the object-centred comforting task by pushing the stuffing back inside was categorized as the same level as verbal comfort.

Interrater reliability (i.e., intraclass correlation) was strong for categorizing level of engagement in all four tasks (object-centred helping: 0.848; social-centred helping: 1.000, object-centred comforting: 0.935, social-centred comforting: 0.956). Because each verbal response could be categorized in one of three ways (i.e., empty, concern, or comfort; children who made no response at all were categorized as “empty” for the interrater analyses), we also examined the raters’ codes for verbal responses separately. In both comforting tasks (very few children responded verbally to either helping task), interrater agreement for distinguishing between the three types of verbal response was moderate (object-centred comforting: *Κ* = 0.660, *p* < 0.001, 78.6% agreement; social-centred comforting: *Κ* = 0.490, *p* < 0.001, 84.4% agreement). Given the fairly high percent agreement between the raters here, these lower Kappa values likely reflect unbalanced marginal totals (Feinstein and Cicchetti, 1990), particularly in the social-centred comforting task, where both raters used the “empty” code far more often than the other two.

The raters also identified *when* the child gave their highest level of response to each of the prosocial tasks (i.e., at the first, second, or third cue). This code was used to create a measure of spontaneity for prosocial acts: helping and comforting responses that occurred at the first cue were considered “most spontaneous,” responses at the second cue were “moderately spontaneous,” and responses at the third cue were “least spontaneous.” Interrater reliability (i.e., intraclass correlation) for identifying when the highest response occurred was strong in all four tasks (object-centred helping: 0.960, social-centred helping: 0.838, object-centred comforting: 0.902, social-centred comforting: 0.751).

Initial review of the data revealed little variance in children’s helping behaviour: most children physically helped the experimenter in both tasks. In contrast, children’s comforting behaviour was far more varied, so we examined it more closely. Specifically, raters categorized children’s verbal responses during the comforting tasks as either object-oriented in content (i.e., the comment was focused on an object relevant to the situation, like the toy or a band-aid), social-oriented (i.e., the comment was focused on the experimenter’s feelings), or irrelevant to the situation (i.e., empty). Interrater agreement for identifying the orientation of verbal responses was moderate in both tasks (object-centred comforting: *Κ* = 0.653, *p* < 0.001, 81.0% agreement; social-centred comforting: *Κ* = 0.394, *p* < 0.001, 81.3% agreement). Once again, these lower Kappa values seem to be caused by unbalanced marginal totals.

We combined the verbal and physical comforting codes in order to identify comforting *strategies* the children used: either object-oriented (e.g., saying “I can get a band-aid” or fixing the broken toy) or social-oriented (e.g., saying “It’s okay” or kissing the experimenter’s knee). Children often made more than one comforting response to a single task, but because we were interested in whether shyness interferes with using social-oriented strategies specifically, we prioritized those responses. That is, *any* social-oriented response earned the social-oriented strategy label, while the object-oriented strategy label was given to children who made *only* object-oriented responses (see the supplemental spreadsheet “Coding Summary for the Comforting Tasks” on our OSF page for further detail).

## Results

We pre-registered the analysis plan for this study on OSF. Changes made to the analysis plan since the pre-registration are outlined in a document on the project’s main page, where we have also made the data and statistical analysis available.

### Prosocial behaviour

Supplementary Table S4 shows how many participants, divided by gender, were given each testing order (A or B) and paired with each experimenter (1 or 2). There were no significant gender or order effects in how children responded to the prosocial tasks. Children’s responses in the two helping tasks and the object-centred comforting task were not affected by the experimenter with whom they were paired, but an experimenter effect was found in the social-centred comforting task. That is, children who were paired with Experimenter 1 (*n* = 23; *Mdn* = 3) responded to her injury with a higher level of engagement, on a scale including both prosocial and non-prosocial responses, than those who were paired with Experimenter 2 (*n* = 19; *Mdn* = 1): *Mann–Whitney U* = 114.50, *p* = 0.007.

#### Comparing performance on the prosocial tasks

**Figure 2:**
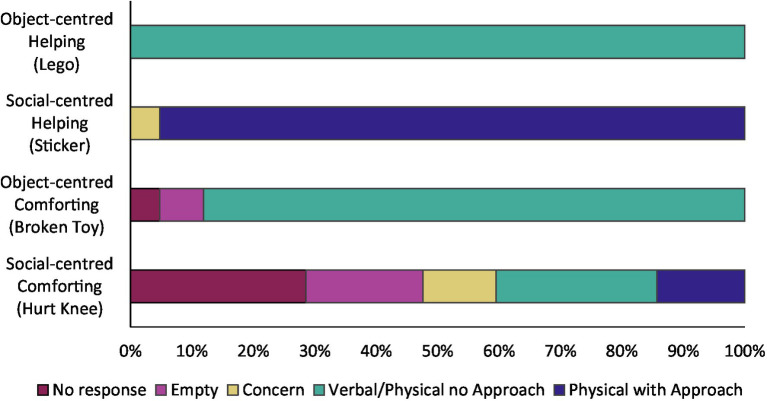
Observed level of engagement in the prosocial tasks. Children’s responses to the four prosocial tasks categorized by level of engagement. Non-prosocial responses include: no response, an empty verbal response, and a concerned response. Prosocial responses include: verbal or physical intervention without approaching the experimenter, and physical intervention with approach. Note that children could not approach the experimenter to help in the object-centred task.

We observed a relatively high frequency of prosocial behaviour in our sample, with all children intervening in at least one of the four tasks. Figure 2 below and Supplementary Table S6 summarize children’s prosocial and non-prosocial responses in each task, arranged by level of engagement. Consistent with past research (e.g., Kienbaum et al., 2001; Dunfield and Kuhlmeier, 2013), one task yielded fewer prosocial acts than the others: only 40.5% of the sample intervened in the social-centred comforting task, compared to 88.1% in the object-centred comforting task, 95.2% in the social-centred helping task, and 100% in the object-centred helping task. Thus, there was little variation in children’s rate of intervention in the two helping tasks. We used Fisher’s exact test to compare children’s interventions in the object-centred and social-centred comforting tasks, but found no significant association between them (*p* = 0.632).

We further categorized children’s comforting by the type of strategy they used in each task. For the object-centred comforting task, where the experimenter was upset about her broken toy, a binomial test found that the number of children who used an object-oriented strategy (e.g., pushing the stuffing back inside; *n* = 26) compared to a social-oriented one (e.g., saying “It’s okay”; *n* = 11) was greater than what would be expected by chance (*p* = 0.020). In contrast, when the experimenter hurt her knee in the social-centred task, the number of children who used an object-oriented strategy (e.g., suggesting they get a band-aid; *n* = 8) compared to a social-oriented one (e.g., giving her knee a kiss; *n* = 9) did not differ (*p* = 1.000).

As detailed in Table 2, children were quite spontaneous when intervening in three of the four tasks: in the object-and social-centred helping tasks, and the object-centred comforting task, more than half of the children who intervened did so at the first cue (i.e., when the experimenter merely stated her problem). Children were less spontaneous in the social-centred comforting task: among the children who intervened when the experimenter hurt her knee, only 35.3% did so at the first cue, while 52.9% did not comfort until the third cue (i.e., when she asked the child directly if they could intervene). We used the Friedman test to examine the spontaneity of children’s prosocial behaviour, which varied significantly across the four tasks: χ^2^(3) = 13.48, *p* = 0.004 (*n* = 16). Post-hoc Wilcoxon signed-rank tests using a Bonferroni corrected *alpha* of 0.008 revealed a significant difference between children’s spontaneity in the object-centred helping and social-centred comforting tasks (*z* = −2.74, *p* = 0.006) and a marginally significant difference between the social-centred helping and social-centred comforting tasks (*z* = −2.60, *p* = 0.009).

**Table 2:** Spontaneity of Prosocial Responses.

	Spontaneity			Total (of 42 participants)
	Least spontaneous (3^rd^ cue)	Moderately spontaneous (2^nd^ cue)	Most spontaneous (1^st^ cue)	
Object-centred Helping	1	15	26	*n* = 42
Social-centred Helping	5	14	21	*n* = 40
Object-centred Comforting	9	7	21	*n* = 37
Social-centred Comforting	9	2	6	*n* = 17

The counts in this table represent the number of children in each task that intervened at the specified cue.

### Parent-reported shyness

Because we calculated a shyness score for each child by summing their mother’s ratings for the 9 items in the Q-Sort shyness subscale, the scores could range from 9 (i.e., a rating of “1” on all items) to 81 (i.e., a rating of “9” on all items). In our sample, shyness scores were less extreme, but still varied: the lowest score was 20 and the highest was 63 (*M* = 37.74; *SD* = 11.77). These scores were approximately normally distributed and did not vary significantly by the gender of the child, the order of the tasks, or the experimenter paired with the child.

### Shyness and prosociality

We tested the relation between children’s shyness and the *spontaneity* of their interventions in all four of the prosocial tasks. When examining *whether* children intervened and *how* they did so, however, we found little variation in either helping task (i.e., most children physically helped the experimenter), so we have focused on the comforting tasks alone for those analyses.

#### Helping: Object-centred and social-centred

Children’s scores on the Q-Sort shyness subscale did not significantly relate to how spontaneously they helped the experimenter in either the object-centred helping task (*n* = 42; Kendall’s *τ_b_* = −0.11, *p* = 0.403) or the social-centred helping task (*n* = 40; Kendall’s *τ_b_* = −0.09, *p* = 0.490).

#### Comforting: Object-centred

In the object-centred comforting task, we used a binomial logistic regression to examine whether scores on the Q-Sort shyness subscale would predict which children comforted. This analysis was not significant: χ^2^(1) = 1.52, *p* = 0.217, *OR* = 0.01. We also used logistic regression to examine whether shyness would predict which of the children who comforted (*n* = 37) used a social-oriented strategy, as opposed to an object-oriented one, and found no relation: χ^2^(1) = 1.91, *p* = 0.167, *OR* = 55.42. We did, however, find a significant relation between shyness and spontaneity of comforting: Kendall’s *τ_b_* = −0.29, *p* = 0.027. That is, among the 37 children who comforted the experimenter when she was upset about her broken toy, higher scores on the shyness subscale were moderately associated with less spontaneous comforting (i.e., the child acted only after multiple cues were given).

#### Comforting: Social-centred

In the social-centred comforting task, shyness scores did not significantly predict which children comforted the experimenter: χ^2^(1) = 0.25, *p* = 0.620, *OR* = 0.68. Shyness also did not predict which of the children who comforted (*n =* 17) used a social-oriented strategy, as opposed to an object-oriented one: χ^2^(1) = 0.90, *p* = 0.342, *OR* = 8.85. Finally, there was no significant relation between shyness and the spontaneity of children’s social-centred comforting (*n* = 17): Kendall’s *τ_b_* = −0.21, *p* = 0.308.

## Discussion

The main goal of our study was to examine whether shy children differ in their motivation to act on behalf of a person in need and/or their understanding of how to do so. Specifically, we examined two factors that may prevent shy children from intervening: social engagement demands and the complexity of the problem to be solved. Because these factors have been conflated in past research (e.g., Beier et al., 2017; Karasewich et al., 2019), we set up our tasks to vary in the source of the experimenter’s problem (i.e., object-vs. social-centred) and the type of intervention required (i.e., helping vs. comforting). Our study provides valuable insights into approaches future research could take to examine individual differences in the prosocial motivation and understanding of young children, both shy and not-shy alike. In particular, our findings present new methodological challenges for researchers interested in separating the effects of social demands and complexity on children’s interventions.

Of the four prosocial tasks that we gave to the 3.5-to 4.5-year-old children in our sample, the social-centred comforting task stood out for its apparent difficulty. Only half as many children intervened when the experimenter was distressed after hitting her knee than in any of the other three tasks. Interventions in the social-centred comforting task were also less spontaneous: around half of the children who comforted did so only at the third cue, while most children required only one cue to act in the other tasks. Even the 16 children who intervened all four times acted less spontaneously in the social-centred comforting task compared to the two helping tasks. These findings are consistent with past research that has found that preschool children help more often than they comfort and are more likely to comfort someone upset over a damaged object than an injury (e.g., Kienbaum et al., 2001; Dunfield et al., 2011; Dunfield and Kuhlmeier, 2013). What is surprising, however, is that responding to the injured experimenter, a complex and highly socially demanding task, was not more difficult for shyer children. The only shyness effect that we found was in the *object*-centred comforting task, where children who scored more highly on the Q-Sort shyness subscale provided comfort less spontaneously. Shyer children were just as likely as less-shy children to be spontaneous when intervening in the object-centred helping, social-centred helping, and social-centred comforting tasks, and shyness had no bearing on whether children comforted or which strategy they used in both comforting tasks.

One explanation for this pattern of results is that we did not have a good representation of shy children in our data. That is, it would be more accurate to consider the subscale we created from the Attachment Q-Sort a measure of *social withdrawal*, because most of its items refer to behaviours associated with shyness rather than feelings (see Supplementary Table S2). It is common for prosocial behaviour researchers to use social withdrawal as a proxy measure for shyness (e.g., Allen et al., 2018; Karasewich et al., 2019). The problem with this method is that shy children are not the only ones who withdraw from social situations. Young children can, for example, be disinterested in other people (i.e., *unsociable*) without also feeling anxious around them (Coplan and Armer, 2007; Rubin et al., 2009). Many of the behaviours described in our shyness subscale could just as likely be displayed by unsociable children, including: avoiding visitors, refusing to talk to strangers, and being slow to smile. It should be noted, however, that three of the five items removed from the subscale for having low inter-item correlation within our sample are very clear measures of unsociability (Waters, 1987): they describe a child who is less interested in people than their own toys, activities, and other things. This suggests that mothers interpreted the nine items that *were* included in the final subscale as distinct from pure social disinterest.

If we do take the shyness subscale at face value, it is interesting to note that no child scored within the top 20%. This restricted range was not due to shyer children being excluded from the analyses; we only excluded one child for not assenting to participate (Supplementary Table S1). Instead, it may be that parents of very shy children are reluctant to bring them to lab-based studies, as they likely feel uncomfortable in unfamiliar social settings. The shyness scores in our sample were, however, just as restricted at the other end of the scale, so it may just be an outcome of the sorting system, with mothers prioritizing other Q-Sort items when forming their “extreme” piles. It is also worth asking whether our testing situation was *too* comfortable. Shy children tend to be anxious around unfamiliar people and intervene less on their behalf, but they can be made to feel more at ease with relatively short, positive interactions (e.g., Young et al., 1999; Allen et al., 2018). Our study included a moderately long warm-up period, which could have made shyer children feel comfortable enough to come to the experimenter’s aid. The experimenter effect we observed in the social-centred comforting task further highlights the dynamic, interpersonal nature of prosociality (Barragan and Dweck, 2014; Martin and Olson, 2015; Kuhlmeier et al., 2020).

Our findings also, necessarily, reflect the design of our tasks. We attempted to use four prosocial problems to disentangle the impacts of social engagement demands and complexity on shy children’s interventions. Our intention was to vary these two factors systematically: to have one task that was both simple and low in social demands (i.e., object-centred helping), one that was both complex and highly demanding (i.e., social-centred comforting), and two that were in-between (i.e., social-centred helping and object-centred comforting). We were most interested in how shyer children would perform in these last, in-between tasks. Would they hesitate to help in a straightforward way if it meant entering someone’s personal space? Would they struggle to figure out how to comfort even when they could do so from a safe distance?

The shyer children in our sample had no problem providing social-centred help. In fact, children’s performance, overall, did not differ between the two helping tasks: they were just as likely to help, and to help spontaneously, when the experimenter had a sticker on her jacket as when she spilled Lego bricks on the floor. We could take this finding to mean that the shyness effects observed in other socially demanding helping tasks (i.e., Beier et al., 2017; Karasewich et al., 2019) were due to their complexity, but that would be a mistake. It is far more likely that our social-centred helping task was not as demanding as we intended. That is, although the experimenter’s problem was within her personal space and thus encouraged children to approach her, it also involved a target object. Shyer children were likely able to focus on the sticker itself and quickly remove it, without feeling taxed by being in close contact with her. This task could thus be considered more similar to the two object-centred tasks than the *social*-centred comforting task. We should, however, question whether the object-centred-tasks were equivalent to *each other* in social demands. Although the spilled Lego bricks and broken toy were both objects a child could focus on while intervening, there was more potential for them to engage with the experimenter while providing comfort. We did not even have a fourth level of engagement in the object-centred helping task; there was no reason for a child to approach the experimenter while they picked up the bricks, especially after she moved to put another toy away. In contrast, offering the experimenter a hug or another form of affection could reasonably make her feel better about her broken toy.

On the surface, the *potential* to be more engaged while intervening does not sound much like a social “demand.” Can we really be sure, though, that all the children in our sample *understood* that they had other options? One of the main reasons we consider comforting problems to be complex for young children is that there is not one clear solution, but many ways they could appropriately respond to the person in distress. Indeed, the children in our sample showed a greater variety of responses to the two comforting tasks, and we were able to categorize their interventions in multiple ways: by form (i.e., verbal or physical), strategy (i.e., object-or social-oriented), and the specific words spoken or actions taken (see the coding summary at our OSF page). Overall, children’s comforting responses in the object-centred task were less engaged than the social-centred task: no child approached the experimenter to comfort her about her broken toy, and the majority used an object-oriented strategy while intervening. We did, however, find that shyer children provided comfort less spontaneously in this task. Again, we could take this finding to mean that complexity is the greatest barrier to shy children’s interventions. This assumes, however, that we were successful in isolating complexity from social demands, but that very complexity may have made it harder for shyer children to initially *see* that they could comfort without a lot of interaction – that trying to fix the toy would be enough to make the experimenter feel better. Future researchers interested in the prosocial decision-making of young children, shy and not-shy alike, should consider how a child’s motivation to intervene may actually *depend* on their understanding of what can be done.

No matter how we explain shyer children’s slow response to the experimenter breaking her toy, it is still puzzling that we did not see a similar shyness effect when she hurt her knee. It is certainly not the case that the object-centred comforting task was more socially demanding. The social-centred task, after all, had no real equivalent to the broken toy, a concrete object sitting right in front of the child – instead, most of the children who used an object-oriented strategy to comfort the injured experimenter did so in an abstract way, like offering to get a band-aid. The prosocial problem here seemed to pull children’s focus to the experimenter herself, and some did engage with her to the highest extent by approaching to kiss her hurt knee. There was an exception: one child focused on the *door* to the room, which he opened wide to show how she could have avoided getting hurt. We did not anticipate this type of response, especially given that the experimenter bumped into the *frame* of the door while it was already ajar. It is important to note, however, that prosocial situations involving an injury *can* include manipulatable objects. Experimenters in other studies, for example, have simulated pinching their fingers in clipboards, dropping baskets on their toes, or hitting their thumbs with toy mallets (Young et al., 1999; Liew et al., 2011; Laible et al., 2021; MacGowan and Schmidt, 2021). All of these comforting problems could be considered object-centred versions of our injury-based task.

A better explanation for the lack of shyness effects in our social-centred comforting task is that children of this age will struggle to comfort an injured person *regardless* of their level of shyness (but see: Young et al., 1999, where shyer toddlers were less likely to comfort a distressed experimenter). After all, only 17 children comforted the experimenter when she hurt her knee, despite most of the sample intervening in all of the other tasks. It seems likely that our two comforting tasks were not equal in complexity. In the social-centred task, children had to think of a way to either soothe the experimenter or heal her injury, but in the object-centred task, they did not need to know how to personally soothe her if they could think of a way her toy may be fixed (Hoffman, 1982; Dunfield, 2014). Thus, while neither problem had an *obvious* solution, as shown by the variety of responses, fixing the broken toy seems to have been a more *accessible* solution. In hindsight, a more equivalent counterpart to our social-centred problem would have been an injury-based task involving a tangible object. For example, MacGowan and Schmidt (2021) examined shy children’s (non-prosocial) empathic responses to an experimenter who “pinched” her finger in a clipboard. At six-years-old, shyer children in their sample were less likely to engage in hypothesis testing (e.g., asking about what happened, pushing down on the clipboard, etc.), which may suggest that they had trouble recognizing why she was upset. In our study, we may have been able to get a more nuanced picture of what children understood about the experimenter hurting her knee if we had examined their non-prosocial behaviours to the same depth as we had their interventions. Another interesting direction for future research would be to examine whether shy children differ in when they see comforting as a normative, obligatory response and how that may relate to their own comforting behaviour (e.g., Paulus et al., 2019; Geraci et al., 2021).

## Conclusion

In this study, we sought to explore how particular aspects of a prosocial situation may affect shy children’s motivation to intervene and their understanding of how to do so. We thus set out to design four prosocial tasks that varied in social engagement demands and complexity. Based on previous research (e.g., Beier et al., 2017; Karasewich et al., 2019), we expected shyer children to be less motivated to intervene in social-centred tasks that encouraged them to engage at a higher level with the person in need. We also expected shyer children, in particular, to have trouble figuring out how to comfort the experimenter when she was distressed. Our findings subverted our expectations in interesting ways. Contrary to our first expectation, the shyer children in our sample did not differ in how often they intervened or their type of response, in any of the four tasks. Regarding the second expectation, we observed that providing social-centered comfort to a recently-introduced adult was challenging for young children in general, regardless of shyness, though shy children did show hesitancy with object-centered comforting.

Although we do need to be cautious in interpreting our social withdrawal-based shyness measure, the results of our study still highlight some of the challenges researchers face in disentangling the effects of social engagement demands from complexity. For example, it is likely that our social-centred helping task was too simple to be socially demanding – shyer children may have been more reluctant to help the experimenter if her problem could not have been solved so quickly. In turn, we suggest that the complexity of the object-centred comforting task made it appear socially demanding – shyer children took longer to intervene because they did not realize, at first, that they *could* act through the broken toy. Finally, few children in our sample comforted the experimenter when she was injured in the social-centred task, implying that this is a difficult problem for shy and not-shy preschoolers alike. Future research could examine shy children’s prosocial motivation and understanding more closely by giving them opportunities to intervene in tasks that are as equivalent as possible and by meticulously measuring their *non*-prosocial responses as well.

## Data availability statement

The original contributions presented in the study are publicly available. This data can be found here: https://osf.io/ykzs9/.

## Ethics statement

The studies involving human participants were reviewed and approved by Queen’s University General Ethics Board. Written informed consent to participate in this study was provided by the participants’ legal guardian/next of kin.

## Author contributions

NB, TK, SP, and VK designed the study and collected the data. TK, CH, and KD conceptualized and completed the analysis and data figures. SP created the method figure. TK, KD, and VK drafted and edited the manuscript. All authors contributed to the article and approved the submitted version.

## Funding

This work was supported by the Social Sciences and Humanities Research Council of Canada through an operating grant awarded to VK (grant number 453-2015-0875) and doctoral fellowship awarded to TK (grant number 752-2019-2576).

## Conflict of interest

The authors declare that the research was conducted in the absence of any commercial or financial relationships that could be construed as a potential conflict of interest.

## Publisher’s note

All claims expressed in this article are solely those of the authors and do not necessarily represent those of their affiliated organizations, or those of the publisher, the editors and the reviewers. Any product that may be evaluated in this article, or claim that may be made by its manufacturer, is not guaranteed or endorsed by the publisher.
