# Supplementary material for: Examining the influence of shyness on children’s helping and comforting behaviour
Source: Front Psychol. 2023 Feb 27;14:1128588. doi: 10.3389/fpsyg.2023.1128588 (PMC10008939; doi:10.3389/fpsyg.2023.1128588)
Supplement: Supplementary file 1 [file Table_1.DOCX]

SUPPLEMENTAL MATERIAL

# Examining the Influence of Shyness on Children’s Helping and Comforting Behaviour

# Participants

Table S1

| *Data Exclusion* | | |
| --- | --- | --- |
| Source | Description | Number of participants excluded |
| Experimenter | Deviation from script  Incorrect order of prosocial tasks | 3  1 |
| Technical | Audio failed to record | 1 |
| Participant | Q-Sort completed incorrectly  Q-Sort not completed  Child did not assent to participate | 2  1  1 |
| Total |  | 9 |

Figure S1

*Sensitivity Power Analysis*


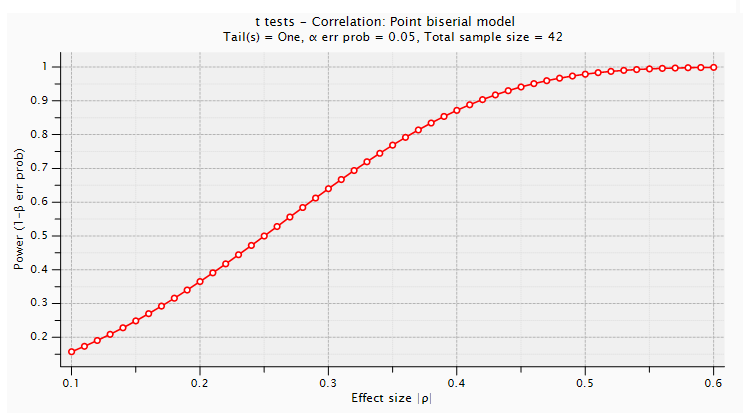


*Note.* Sensitivity power curve showing the power that our sample size (*N* = 42) would have to detect a range of effect sizes in a correlation analysis, with an alpha of 0.05. Power caps off here at an effect size of 0.53.

# Attachment Q-Sort

## Sorting Piles

Figure S2

*Attachment Q-Sort Piles*

*somewhat*

*uncharacteristic*

*quite*

*uncharacteristic*

1

2

3

4

5

6

7

8

9

*quite*

*characteristic*

*somewhat*

*characteristic*

*extremely*

*uncharacteristic*

*extremely*

*characteristic*

*neutral*

*fairly*

*uncharacteristic*

*fairly*

*characteristic*

*Note.* Mothers had to sort each card into one of 9 piles, based on how well its description matched their own child’s typical behaviour. Each pile had to contain 10 cards at the end of the sort.

The Attachment Q-Sort can be used to calculate scores of attachment security by correlating the rater’s (in this case, each child’s mother’s) choice of pile for each item to a ‘criterion sort,’ which was constructed by averaging the ratings of eight experts for a hypothetical ‘most securely attached’ child (Waters & Deane, 1985; Waters, n.d.). Using this method, security scores can range from ‑1.00 to 1.00, with larger positive correlation coefficients indicating more secure attachment. In our sample, we found attachment to be restricted to higher security: scores ranged from .20 to .66, with a mean of .47 and a standard deviation of .11. Due to this restricted range, we have not included attachment security as part of our analyses.

## Shyness Subscale

Table S2

| *Q-Sort Shyness Subscale* | |
| --- | --- |
| Items in the scale | |
| 3.  7.  12.  15.  48.  49.  50.  66.  78. | When he is upset or injured, child will accept comforting from adults other than mother (R)†  Child laughs and smiles easily with a lot of different people (R)  Child quickly gets used to people or things that initially made him shy or frightened him (R)  Child is willing to talk to new people, show them toys, or show them what he can do, if mother asks him to (R)  Child readily lets new adults hold or share things he has, if they ask to (R)  Child runs to mother with a shy smile when new people visit the home  Child’s initial reaction when people visit the home is to ignore or avoid them, even if he eventually warms up to them  Child easily grows fond of adults who visit his home and are friendly to him (R)  Child enjoys being hugged or held by people other than his parents and/or grandparents (R) |
| Items removed from the scale | |
| 5.  56.  58.  67.  76. | Child is more interested in people than in things (R)  Child becomes shy or loses interest when an activity looks like it might be difficult  Child largely ignores adults who visit the home. Finds his own activities more interesting  When the family has visitors, child wants them to pay a lot of attention to him (R)  When given a choice, child would rather play with toys than with adults |
| †(R) denotes items that were reverse-coded  Item #56 does not refer to either social withdrawal or its opposite, but was included as a possible item in the shyness subscale because its use of the word ‘shy’ could have influenced how mothers sorted it. It was not a surprise to find that this item needed to be removed.  Item #12 should actually be considered a measure of *behavioural inhibition*, because it refers to ‘things’ as well as ‘people’. Removing this item from the scale, however, does not significantly change our results (see below). | |

## Removing Behavioural Inhibition from the Q-Sort Analyses

There is clear overlap between behavioural inhibition and shyness in that both inhibited and shy children tend to withdraw around unfamiliar people, but these constructs should be treated as distinct. *Behavioural inhibition* goes beyond the social context: it is a tendency for a young child to withdraw from *all* forms of novelty, including unfamiliar objects, events, and settings, as well as people (Calkins & Fox, 1992; Kagan & Snidman, 1991). Shyness, in turn, goes beyond social novelty: shy children will act withdrawn whenever they feel anxious, even around people they know (Crozier & Badawood, 2009; Gazelle & Ladd, 2003). It is important, then, for us to assess whether the behavioural inhibition item (#12) that was included in our shyness subscale had any impact on our results.

Eight items remain in the Q-Sort shyness subscale when the behavioural inhibition item is removed, so scores can now range from 8 to 72. Internal consistency was slightly lower than the original (Cronbach’s *alpha* = .780). In our sample, the lowest score on the eight-item subscale was 16 and the highest was 54 (*M* = 33.29, *SD* = 10.46). These scores were approximately normally distributed and there were no gender, order, or experimenter effects.

Shyness measured with the eight-item subscale did not predict which children comforted in the object-centred task (*n* = 42, χ^2^(1) = 1.37, *p* = .241, *OR* = 0.01) or the social-centred task (*n* = 42, χ^2^(1) = 0.50, *p* = .478, *OR* = 0.56). It also did not predict which of the children who comforted used a social-oriented strategy, as opposed to an object-oriented one, in either task (object-centred: *n* = 37, χ^2^(1) = 1.62, *p* = .203, *OR* = 25.83; social-centred: *n* = 17, χ^2^(1) = 1.35, *p* = .246, *OR* = 15.80). Shyness here was not related to how spontaneously children intervened in the object-centred helping task (*n* = 42, *τ_b_* = -.11, *p* = .395), the social-centred helping task (*n* = 40, *τ_b_* = -.07, *p* = .567), or the social‑centred comforting task (*n* = 17, *τ_b_* = ‑.22, *p* = .287). It was, however, significantly related to spontaneity in the object-centred comforting task (*n* = 37, *τ_b_* = ‑.27, *p* = .043), with higher scores on the eight-item subscale being moderately associated with less spontaneous comforting. Thus, we replicated all of the results we found when we examined how children’s prosocial behaviour related to their shyness measured with the original, nine-item, subscale.

# Counterbalancing

Table S3

| *Task Orders* | | | | |
| --- | --- | --- | --- | --- |
| Order A | |  | Order B | |
| 1. Puzzle  2. Tower  3. Lego  4. Memory  5. Hurt Knee  6. Tic-Tac-Toe  7. Sticker  8. Broken Toy | *delay game*  *delay game*  *object-centred helping*  *delay game*  *social-centred comforting*  *delay game*  *social-centred helping*  *object-centred comforting* |  | 1. Puzzle  2. Hurt Knee  3. Tower  4. Lego  5. Memory  6. Broken Toy  7. Tic-Tac-Toe  8. Sticker | *delay game*  *social-centred comforting*  *delay game*  *object-centred helping*  *delay game*  *object-centred comforting*  *delay game*  *social-centred helping* |

Table S4

| *Counterbalancing* | | | |
| --- | --- | --- | --- |
| Order | Experimenter 1 | Experimenter 2 | Total |
| A: Helping task first | male: 6  female: 6 | male: 5  female: 5 | 22 |
| B: Comforting task first | male: 3  female: 8 | male: 5  female: 4 | 20 |
| Total | 23 | 19 | 42 |

Table S5

| *Full Procedure for the Prosocial Tasks* | |
| --- | --- |
| Task | Cue† |
| Object-centred Helping | 1. The experimenter ‘accidentally’ spilled a bucket of Lego bricks on the ground, then said, “Whoops, I spilled the bricks!” 2. She put two of the bricks back into the bucket and reached for some further away, while saying, “I can’t reach them all!” 3. She asked the child, “Is there something you can do?”   If the child did not intervene within 15 seconds or only picked up some of the bricks, the experimenter moved to pick up the rest herself and said, “It’s okay, I’ve got them.”  If the child did start to help, she left them to pick up the rest of the bricks alone, saying, “I’ll just put the puzzle away.” |
| Social-centred Helping | 1. The experimenter put on a jacket because she felt “a little chilly.” She then ‘realized’ that there was a square sticker stuck to the back of the jacket and said, “Whoops, I think I have something on my back!” 2. She ‘struggled’ to reach for the sticker and said, “I can’t reach it!” 3. She asked the child, “Is there something you can do?”   If the child did not intervene within 15 seconds or only made a verbal helping response (e.g., identifying where the sticker was on her back), the experimenter took off the sticker herself and said, “It’s okay, I’ve got it.” |
| Object-centred Comforting | 1. The experimenter brought out a toy dog to show the child, which she described as her favourite toy. She then ‘noticed’ a rip in the dog’s paw and said, “Oh no! My dog! I ripped my doggy!” She put the toy on the table in front of the child and covered her face with her hands. 2. She said, “Oh no, I’m really sad!” 3. She asked the child, “Is there something you can do?”   If the child did not intervene within 15 seconds or only made a verbal comforting response (e.g., suggesting they ask the child’s parents to sew the rip), the experimenter ‘realized’, “Oh we can push the stuffing back inside! See it’s all better now,” and put the toy away. |
| Social-centred Comforting | 1. The experimenter went into an adjoining room to get more toys and ‘accidentally’ hit her knee on the doorframe coming back. She said “Ouch” in a pained tone of voice while sitting down and rubbing her knee, then said, “Oh, my knee! I banged my knee!” 2. She said, “Ouch, that really hurts!” 3. She asked the child, “Is there something you can do?”   If the child did not intervene within 15 seconds or only made a verbal comforting response (e.g., suggesting they get a bandage), the experimenter rubbed her knee again and said, “Oh, I am all better now! I just had to rub it.”  If the child offered to give physical comfort but did not move to do so, she asked, “Can you give it a try?” to prompt them further. |
| †Each cue was separated by an interval of approximately five seconds. The task ended when the child intervened at the highest level of engagement (i.e., *helping* to pick up the target objects; approaching the experimenter to give her physical *comfort*) or after 15 seconds had elapsed. | |

# Responses to the Prosocial Tasks

Table S6

| *Observed Responses in the Prosocial Tasks* | | | | | |
| --- | --- | --- | --- | --- | --- |
|  | Non-Prosocial Responses | | Prosocial Responses | |  |
| Task | Response Type | *n* | Response Type | *n* |  |
| Object-Centred Helping |  |  | Physical help | 42 |  |
| Social-Centred Helping | Concern | 2 | Physical help w/ approach | 40 |  |
| Object-Centred Comforting | No response  Empty verbal | 2  3 | Verbal comfort  Fixed toy  Verbal comfort + fixed toy | 23  5  9 |  |
| Social-Centred Comforting | No response  Empty verbal  Concern | 12  8  5 | Verbal comfort†  Physical comfort w/ approach | 11  6 |  |
| †One child who offered verbal comfort in the social-centred comforting task also acted physically, but did not approach the experimenter: he opened the door to the next room wide to show her what she ‘should’ve done’ to avoid hitting the frame. We did not expect this type of physical comfort, but like fixing the toy in the object-centred comforting task, we considered it to be at the same level of engagement as verbal comfort. We also categorized this response as object-oriented (Epstein & Baker, 2019; Henderson & Hollingworth, 1999). | | | | | |

# Speech as a Measure of Shyness

In addition to our parent-report measure of shyness, we created an observational measure of shyness by coding children’s speech behaviour during a warm-up period at the start of the study. This measure was based on prior research that found shy children to speak less during social interactions and, in particular, to make fewer spontaneous utterances than not-shy children (e.g., Crozier & Badawood, 2009; Rezendes et al., 1993; but see: Coplan et al., 2004).

## Coding of the Child’s Speech

In the fall of 2020, research assistants transcribed the conversations between the experimenter and each child during the puzzle delay game for the purpose of coding the child’s speech for shy behaviours. The transcribers added a new line for each utterance that the experimenter and child made, with the boundaries of an utterance defined as 3 seconds of silence by the speaker.

We chose to code speech behaviour in the puzzle delay game, specifically, for a few reasons. First, the puzzle was the very first activity children performed in the study and acted as a warm-up period. Children’s behaviour during the puzzle game thus reflected their initial level of comfort in the testing situation, whereas their behaviour in the later games would have been contaminated by their developing relationship with the experimenter. The quality and length of the conversations observed in the puzzle game also most suited our needs. Children spent much longer making the puzzle with the experimenter than they did playing two of the other games: the average length of the puzzle was 6.15 minutes (*SD* = 1.96; range: 3.08-10.90), while the average length of the tower game was 1.68 minutes (*SD* = 0.70; range: 0.75-4.00) and the average length of tic-tac-toe was 1.61 minutes (*SD* = 0.76; range: 0.42-3.77). Although children played the memory game for longer (*M* = 4.56 minutes; *SD* = 1.57; range: 0.57-10.70),, our transcribers noted that the majority of utterances children made were merely labelling the animals they saw on the cards (e.g., “A cow”; “Polar bear and a goose”). Conversation during the puzzle was more varied and often went beyond deciding where to put the pieces to discussing the television show Paw Patrol (which was pictured on the puzzle) and other topics (e.g., “[My teacher] has a dog and it’s really friendly”).

Transcripts of the puzzle game were coded by two independent raters who overlapped on over 25% of the sample, which was used to calculate interrater agreement. Disagreements were resolved by the principal investigator. The raters categorized each of the child’s utterances as either: 1) responsive (i.e., the child was following the experimenter’s lead ), 2) initiating (i.e., the child moved the conversation in a new direction), or 3) irrelevant (i.e., the child’s utterance contained only a gesture, self-talk, or thinking sounds, or had been marked as unclear by the transcriber). The raters agreed on 83.5% of their ratings (*Κ* = .733, *p* < .001).

An observed shyness score was calculated for each child by summing the number of responsive utterances they made during the warm-up ‘puzzle’ game and dividing by their total number of utterances (i.e., responsive + initiating; utterances that were coded as ‘irrelevant’ for our purposes were excluded from the calculation). This score was made into a percentage by multiplying it by 100, for ease of interpretation. Low scores on this measure would indicate that the child spent most of the conversation taking initiative when speaking to the experimenter and would be classified as ‘not shy’, while high scores would indicate that the child spent most of the conversation merely responding to the experimenter’s comments and would be classified as ‘very shy’. In our sample, observed shyness scores ranged from 13.33% to 85.71% (*M* = 32.90; *SD* = 14.49). Observed shyness did not vary significantly based on the gender of the child, the order of the prosocial tasks, or the experimenter paired with the child.

### Observed vs. Parent-reported Shyness

Children’s observed shyness was not related to their parent‑reported shyness (i.e., scores on the Q-Sort shyness subscale): *r* = .14, *p* = .379, *N* = 42. Here, we are reporting the relation between scores on the Q-Sort shyness subscale and a log transformation of observed shyness scores, because the latter distribution was found to be positively skewed and peaked (skew = 4.61, kurtosis = 5.49; both above the criterion of +/- 2.33: Tabachnick & Fidell, 2013). The transformed observed shyness scores had an approximately normal distribution (skew = 0.93, kurtosis = 0.38).

## Coding of the Experimenter’s Speech

We decided to examine the experimenter’s own speech behaviour during the puzzle game to determine what effect she may have had on children’s observed shyness. The same coding process was used for the experimenter’s speech, with two independent raters, overlapping on 25% of the sample, categorizing her speech as either: 1) responsive (i.e., the experimenter was following the child’s lead with her next utterance), 2) initiating (i.e., the experimenter moved the conversation in a new direction), or 3) irrelevant (i.e., the experimenter’s utterance contained only a gesture, thinking sounds, or had been marked as unclear by the transcriber). The raters agreed on 79.8% of their ratings (*Κ* = .626, *p* < .001).

A ‘taking the lead’ score was calculated for both experimenters in their interactions with the children in their half of the sample (*n* = 23; *n* = 19) by summing the number of *initiating* utterances they made in the warm-up game and dividing that by their total number of utterances (i.e., responsive + initiating, with irrelevant utterances excluded from the calculation). The score was made into a percentage by multiplying it by 100. Lower ‘taking the lead’ scores would indicate that the experimenter spent most of the conversation allowing the child to initiate conversation rather than leading herself, while higher scores would indicate that the experimenter mostly tried to lead the conversation. In our sample, ‘taking the lead’ scores ranged from 33.33% to 96.97% (*M* = 70.30, *SD* = 15.25).

### Experimenter Differences

In our [pre-registration](https://osf.io/y25a8?view_only=521e5851131d4df68c741ed5ad79cc5e) for this study, we reported an independent t-test calculated to assess whether the two individuals who acted as the experimenter differed in how much the spoke during the warm-up period. This initial test was not statistically significant, but we felt that the difference was large enough to make interpretation of children’s own speech behaviour difficult (*t*(40) = 1.78, *p* = 0.82), so we decided to consider the observed shyness measure as exploratory only. At the time, we had not yet coded the experimenters’ speech, so this initial test included their irrelevant utterances as well. When we re-ran the t-test with only the experimenters’ utterances that would be used in the following analyses (i.e., initiating and responsive) we found a similar result: *t*(40) = 2.25, *p* = .030, Cohen’s *d* = 0.74. Note that here, we are reporting a log transformation of the number of utterances each experimenter made per second, because we found Experimenter 2’s distribution to be positively skewed and peaked (skew = 3.50, kurtosis = 4.19; both above the criterion of +/- 2.33: Tabachnick & Fidell, 2013). After the transformation, both experimenters had approximately normal distributions (Experimenter 1: skew = ‑2.01, kurtosis = 0.94; Experimenter 2: skew = 1.33, kurtosis = 0.82).

We also tested whether the experimenters differed in how often they took the lead in the warm-up conversations. An independent t-test did not find a significant difference in the experimenters’ ‘taking the lead’ scores: *t*(40) = 1.43, *p* = .160, Cohen’s *d* = 0.45. We did, however, find that the experimenters differed in whether their speech behaviour related to the child’s own. For Experimenter 1, children’s observed shyness (log transformed) was significantly related to how often she took the lead in conversations: *r* = .59, *p* = .003, *n* = 23. For Experimenter 2, children’s observed shyness (log transformed) was *not* significantly related to how often she took the lead: *r* < .01, *p* = .989, *n* = 19. Given this discrepancy, we have added the experimenter paired with each child as a second predictor in all of the analyses in the next section.

## Observed Shyness and Prosociality

In the following analyses we examine the relation between children’s level of shyness and their performance in the four prosocial tasks. Because there was little variation in *whether* children helped and *how* they did so in both helping tasks, we have focused on the comforting tasks for those analyses. We were able to examine the relation between shyness and spontaneity in all four prosocial tasks, however, because children did vary in how spontaneously they helped.

### Helping: Object-centred

In the object-centred helping task, neither observed shyness scores nor the experimenter paired with the child significantly predicted how spontaneously children helped the experimenter (*n* = 42): observed shyness: χ^2^(2) = 5.01, *p* = .082; experimenter: χ^2^(2) = 1.49, *p* = .476. Here, we are reporting the results of a multinomial logistic regression, because our data violated the assumption of proportional odds that is required for ordinal regression (i.e., the test of parallel lines: χ^2^(2) = 8.53, *p* = .014). The results of this multinomial regression should be interpreted cautiously, as it involves many observed cells with zero frequencies.

### Helping: Social-centred

In the social-centred helping task, we used ordinal regression to examine whether children’s observed shyness scores or the experimenter paired with them would predict how spontaneously children provided help (*n* = 40). Neither predictor was significant in this analysis: observed shyness: χ^2^(1) = 0.01, *p* = .905, *OR* = 1.00; experimenter: χ^2^(1) = 1.65, *p* = .199, *OR* = 2.27.

###

### Comforting: Object-centred

In the object-centred comforting task, we used a binomial logistic regression to examine whether children’s observed shyness scores or the experimenter paired with them would predict which children comforted the experimenter. Neither predictor was significant in this analysis; observed shyness: χ^2^(1) = 0.66, *p* = .416, *OR* = 1.53; experimenter: χ^2^(1) = 1.18, *p* = .278, *OR* = 3.71. Observed shyness and the experimenter also did not predict which of the children who comforted (*n* = 37) used a social-oriented comforting strategy, as opposed to an object-oriented one: observed shyness: χ^2^(1) = 0.38, *p* = .541, *OR* = 0.68; experimenter: χ^2^(1) = 1.54, *p* = .215, *OR* = 0.37. Finally, in an ordinal regression examining how spontaneously children comforted (*n* = 37), neither observed shyness nor the experimenter paired with the child was a significant predictor: observed shyness: χ^2^(1) = 0.31, *p* = .575, *OR* = 0.99; experimenter: χ^2^(1) = 0.75, *p* = .388, *OR* = 1.77

### Comforting: Social-centred

In the social-centred comforting task, a binomial logistic regression found that which children provided comfort was not predicted by their observed shyness scores (χ^2^(1) = 2.00, *p* = .157, *OR* = 1.85), but was *marginally* predicted by the experimenter with whom they were paired (χ^2^(1) = 3.63, *p* = .057, *OR* = 0.25). A separate logistic regression examining whether observed shyness and the experimenter predicted which of the children who comforted (*n* = 17) used a social-oriented comforting strategy, as opposed to an object-oriented one, was also not significant; observed shyness: χ^2^(1) = 0.01, *p* = .916, *OR* = 0.88; experimenter: χ^2^(1) = 1.23, *p* = .268, *OR* = 0.22. Finally, an ordinal regression found neither observed shyness nor the experimenter paired with the child to predict how spontaneously children comforted (*n* = 17): observed shyness: χ^2^(1) = 0.23, *p* = .630, *OR* = 1.02; experimenter: χ^2^(1) = 0.91, *p* = .340, *OR* = 0.34.

## Discussion of Speech Analyses

Children who mostly responded to the experimenter’s comments during the warm-up game, rather than initiating conversation themselves, were just as likely as less-shy children to comfort the experimenter in the object- and social-centred tasks, to use a social‑oriented strategy when comforting in both tasks, and to respond spontaneously in all four prosocial tasks. This is similar to our findings in the main text, where shyness was measured with the Q‑Sort shyness subscale, with the exception that parent-reported shyness *did* relate to how spontaneously children comforted in the object-centred comforting task.

It is important to note that the observed shyness measure was created post-hoc, after the data had already been collected, so he two individuals acting as the experimenter were not given any directions about what they should say during the warm-up game. When we examined their own speech behaviour during the warm-up period, we found that they differed in how often they spoke and how their speech behaviour related to the child’s own. For the experimenter who spoke more, how often she took the lead was directly related to children’s observed shyness. This finding makes intuitive sense, given that it is polite for an individual to make a responsive comment whenever someone initiates conversation, however we used a fairly liberal definition of ‘initiating’ when coding: if any part of the child’s utterance moved the conversation forward, even if it was on the same general topic as the experimenter’s last comment, then that utterance was labelled ‘initiating.’ So, we would not necessarily expect to see the number of initiating and responsive comments made by the experimenter and child to show an inverse relation. That children were more responsive when Experimenter 1 took the lead may instead suggest that there were also other qualities of the warm-up conversations that differed between the two experimenters.

Another consequence of adding the observed shyness measure after data collection was that we did not have control over the amount of time the experimenter and child spent playing the warm-up game. For this reason, we calculated observed shyness as the percentage of each child’s utterances that were responsive, rather than using raw scores. Although this choice did make interpreting the observed shyness scores easier in some respects, it does create a problem in cases where a child did not speak very much during the warm-up period. If a child only spoke a few utterances for the entire game and those utterances happened to be mostly initiating, does that really mean that the child was not shy? Does a lack of chattiness mean that a child was actually very shy (e.g., Rezendes et al., 1993; Schneider, 1999) or could it mean instead that they were too focused on putting together the puzzle to hold a conversation? Although there was a wide range in the number of utterances children spoke during the puzzle game (i.e., from only 5 utterances to 73), there was no clear cut-off point at which we could exclude children for not speaking ‘enough’ to get a more accurate shyness score.

Finally, it is of note that the scores on our two shyness measures – speech during the warm-up and the Q-Sort shyness subscale – did not relate to each other. Although it is not uncommon for parent-report and observational measures to differ (possibly due to biases in parent-reporting or to short-term causes of behaviour change, like a child’s mood: Eisenberg, 1992; Seifer et al., 2004), this finding adds to our concern that our speech measure may not have accurately reflected the shyness of the children in our sample.

# References

Calkins, S. D., & Fox, N. A. (1992). The relations among infant temperament, security of attachment, and behavioral inhibition at twenty-four months. *Child Development*, *63*, 1456-1472.

Coplan, R. J., Prakash, K., O’Neil, K., & Armer, M. (2004). Do you “want” to play? Distinguishing between conflicted shyness and social disinterest in early childhood. *Developmental Psychology*, *40*(2), 244-258.

Crozier, W. R., & Badawood, A. (2009). Shyness, vocabulary, and children’s reticence in Saudi Arabian preschools. *Infant and Child Development*, *18*, 255-270. <https://doi.org/10.1002/icd.623>

Eisenberg, N. (1992). *The caring child*. Cambridge, MA: Harvard University Press.

Epstein, R. A., & Baker, C. I. (2019). Scene perception in the human brain. *Annual Review of Vision Science*, *5*, 373-397. <https://doi.org/10.1146/annurev-vision-091718-014809>

Gazelle, H., & Ladd, G. W. (2003). Anxious solitude and peer exclusion: A diathesis-stress model of internalizing trajectories in childhood. *Child Development*, *74*(1), 257-278.

Henderson, J. M., & Hollingworth, A. (1999). High-level scene perception. *Annual Review of Psychology*, *50*, 243-271.

Kagan, J., & Snidman, N. (1991). Temperamental factors in human development. *American Psychologist*, *46*(8), 856-862.

Rezendes, M., Snidman, N., Kagan, J., & Gibbons, J. (1993). Features of speech in inhibited and uninhibited children. In K. H. Rubin & J. B. Asendorpf (Eds.), *Social withdrawal, inhibition, and shyness in childhood* (pp. 177-187). Hillsdale, New Jersey: Lawrence Erlbaum Associates.

Schneider, B. H. (1999). A multimethod exploration of the friendships of children considered socially withdrawn by their school peers. *Journal of Abnormal Child Psychology*, *27*(2), 115-123.

Seifer, R., Sameroff, A., Dickstein, S., Schiller, M., & Hayden, L. C. (2004). Your own children are special: Clues to the sources of reporting bias in temperament assessments. *Infant Behavior & Development*, *27*, 323-341. <https://doi.org/10.1016/j.infbeh.2003.12.005>

Tabachnick, B. G., & Fidell, L. S. (2013). Cleaning up your act: Screening data prior to analysis. *Using multivariate statistics* (6^th^ ed.). (pp. 73). Toronto, Canada: Pearson.

Waters, E. (n.d.). Assessing secure base behavior and attachment security using the q-sort method. *Attachment: Theory & Research @ Stony Brook.* [http://www.psychology.sunysb.edu/attachment/measures/content/aqs_method.html](about:blank)

Waters, E., & Deane, K. E. (1985). Defining and assessing individual differences in attachment relationships: Q-Methodology and the organization of behavior in infancy and early childhood. *Monographs of the Society for Research in Child Development*, *50*, 41-65.
